# Long-Term Remodeling of Aortoiliac Vessels After Standard EVAR, the Reality to Be Considered

**DOI:** 10.3390/jcm14165626

**Published:** 2025-08-08

**Authors:** Apostolos G. Pitoulias, Matthaios G. Pitoulias, Dimitrios A. Chatzelas, Loukia A. Politi, Efthymios Beropoulis, Mathias Wilhelmi, Georgios A. Pitoulias

**Affiliations:** 1 Division of Vascular Surgery, 2nd Department of Surgery, Faculty of Medicine, “G. Gennimatas” General Hospital of Thessaloniki, School of Health Sciences, Aristotle University of Thessaloniki, 54124 Thessaloniki, Greece; appitoulias@yahoo.com (A.G.P.); man.pitoulias@gmail.com (M.G.P.); eletterbox_dc@outlook.com (D.A.C.); loukiapo@hotmail.com (L.A.P.); eberopoulis@gmail.com (E.B.); 2 Department of Vascular and Endovascular Surgery, St. Bernward Hospital, 31134 Hildesheim, Germany; m.wilhelmi@bernward-khs.de; 3 Lower Saxony Center for Biomedical Engineering, Implant Research and Development (NIFE), Stadtfelddamm 34, 30625 Hannover, Germany

**Keywords:** EVAR, long-term, aortoiliac remodeling, migrations, sac regression

## Abstract

**Background/Objectives:** The aim of our study was to document and analyze the long-term geometric alterations that occur in the infrarenal aorta and iliac arteries over time after a successful elective standard endovascular abdominal aneurysm repair (EVAR) as well as to investigate the potential relationship of aortoiliac remodeling with the long-term complications of EVAR. **Methods:** The prospectively collected clinical and computed tomography angiography (CTA) data from 168 patients treated with elective standard EVAR between 2013 and 2018 were retrospectively analyzed. Follow-up assessments were performed at 1, 24, and 60 months postoperatively. Primary anatomical variables included 11 measurements: total right and left aortoiliac lengths, infrarenal aortic length, right and left aortoiliac angles on the frontal CTA plane, right and left intra-iliac angles, inter-iliac angle, infrarenal aortic body angle on the sagittal CTA plane, and right and left aortoiliac angles on the sagittal CTA plane. Secondary variables were the mean percentage changes in anatomical measurements between the follow-up time intervals. The primary clinical endpoint was the occurrence of any complication related (ARC) to the index EVAR or reinterventions. Secondary endpoints included any graft migration (AM) observed in proximal aortic or distal iliac sealing zones, and failure of aneurysm sac regression (FSR) or an increase in sac diameter by >5 mm. Six different bifurcated endografts were used. For subgroup analysis, the primary differentiating feature among grafts was the presence or absence of suprarenal fixation with hooks. **Results:** Median follow-up was 77 months, with an interquartile range (IQR) of 24.0 months. Observed EVAR-related mortality was 2.4%. Twenty-seven (16.1%) ARC events occurred, and migration was detected in 21 (12.5%) patients, combined with endoleak in 20 of them. The incidence of FSR was 43.5%, and approximately a third of ARCs and AMs occurred after the 60th month of follow-up. Across all measured lengths and the inter-iliac angle on the frontal CTA plane, a significant increase was observed, while all other angles demonstrated a significant decrease over time. The pattern of aortoiliac remodeling followed a linear progression for the first 24 months, transitioning to either a quadratic or cubic trend by the 60-month mark. Linear regression analysis revealed that an excessive increase in length variables was significantly associated with lower AAA sac regression rates. Furthermore, multivariate analysis identified that suprarenal fixation with hooks was the only factor associated with a reduced likelihood of AMs and a five-fold decrease in FSRs. **Conclusions:** Despite a fully successful EVAR, significant aortoiliac geometrical remodeling is evident over time. Extensive remodeling of aortoiliac lengths appears to be associated with lower rates of AAA sac regression. Suprarenal proximal aortic fixation with hooks may serve as a protective mechanism, reducing the likelihood of long-term complications. Life-long follow-up remains an essential measure for early detection of long-term EVAR failures.

## 1. Introduction

Repair of abdominal aortic aneurysm (AAA) using standard endovascular techniques (EVAR), has remained one the main interests of the vascular scientific community and industry for decades. Today, EVAR has become the gold-standard treatment option for the majority of AAAs. Over the years, EVAR has demonstrated remarkable advancements in techniques, endografts, and materials, overcoming many initial limitations and thus offering expanded applicability to the vast majority of AAAs [1]. However, randomized studies and systematic reviews report reintervention rates of 20–30%, raising concerns about the long-term effectiveness and durability of EVAR, particularly in younger patients, where its use remains controversial and subject to skepticism [2,3,4]. As type Ia endoleaks are the primary reason for reintervention, much of the current research has focused on proximal aortic neck (PAN) remodeling [5,6,7,8]. Despite these earlier assumptions, emerging evidence suggests that EVAR failure may not be solely due to PAN compromise. Instead, it may reflect broader, progressive remodeling and geometric alterations throughout the aortoiliac anatomy. [9,10,11]. Driven by the limited evidence on the long-term impact of iliac vessels and the distal landing zone on EVAR outcomes, we undertook this study to address this gap.

The aim of this study is to document and evaluate post-EVAR morphological alterations in terms of the length and angulation geometry of the aorta and iliac arteries, and to assess the implications of the overall aortoiliac remodeling in the development of complications and adverse events.

## 2. Methods

Between 2013 and 2018, 231 consecutive patients with a true fusiform AAA were treated at a single tertiary university hospital using elective standard EVAR. All procedures were performed by a single vascular surgeon following a consistent protocol for the preoperative planning, operative technique, and follow-up. All computed tomography angiography (CTA) and clinical data were collected prospectively. The research protocol for this non-interventional prospective cohort study was approved by our institutional ethics committee and was in accordance with the principles of the Declaration of Helsinki. All patients provided written informed consent prior to EVAR, permitting the use of their anonymized medical data for follow-up analysis and publication of study results.

Our elective standard EVAR protocol included all asymptomatic AAAs with a maximum sac diameter ≥ 55 mm, or ≥50 mm in rare cases with rapid aneurysm expansion (>5 mm/year) and/or female gender. Additionally, EVAR was offered to patients over 65 years of age or in younger patients with severe comorbidities and contraindications for open surgical repair. Anatomo-morphological exclusion criteria for elective standard EVAR included patients with hostile anatomy. To define hostile anatomy, the following were considered: hostile features of the PAN, including length < 12 mm, width or bulge > 30 mm, angulation > 60°, conical anatomy and circumferential thrombus or calcification > 50%, and a hostile distal iliac neck anatomy with a diameter ≥ 24 mm [6,7,8,9,10,11,12]. Eligibility for inclusion in the final analysis was based on two criteria. The first criterion was technical success at 30 days post EVAR, defined as survival at 30 days, with stent graft patency and absence of type I, III, or IV endoleaks; graft migration; or any other EVAR-related complication, as confirmed by CTA. The second criterion was the completion of at least 60 months of CTA follow-up. In accordance with our protocol, all patients who experienced EVAR-related adverse events were included in the analysis, regardless of follow-up duration. Clinical exclusion criteria included the presence of malignancy; autoimmune, inflammatory, or other serious systemic disease; and the presence of pre-existing or new-onset chronic renal failure with an eGFR < 45 mL/min/1.73 m^2^, limiting the use of iodinated contrast in long-term CTA surveillance. Furthermore, patients who died during follow-up from causes unrelated to the index EVAR or secondary procedures as well as patients lost to follow-up for unknown reasons were excluded. Finally, patients treated with an abdominal endograft, comprising fewer than ten total cases, were also excluded.

In all patients, a contemporary CE-marked bifurcated self-expanding abdominal endograft was implanted. The following 6 bifurcated self-expanding stent-graft devices were used: Endurant^TM^ ΙΙ (Medtronic, Santa-Rosa, CA, USA), Treo^®^ (Terumo Aortic, Bolton Medical Inc, Sunrise, FL, USA), E-tegra^®^ (Artivion, Kennesaw, GA, USA), Anaconda^TM^ (Terumo Aortic, Bolton Medical Inc, Sunrise FL, USA), AFX^®^ 2 (Endologix, Irvine, CA, USA), and Ovation^TM^ (Endologix, Irvine, CA, USA). The fabric liner in the AFX^®^2 and Ovation^TM^ devices is made from ePTFE and that in the remaining four (Endurant^TM^ ΙΙ, Anaconda^TM^, Treo^®^, and E-tegra^®^) is made from Dacron (woven polyester).

All patients were enrolled in a standardized and rigorous follow-up protocol with clinical evaluation and color duplex ultrasound (cDUS) examination every six months, as well as with CTA imaging after the first postoperative month to verify the 30-day technical success and at 1, 2, and 5 years in order to detect any follow-up adverse outcomes, including endoleak, migration, stent graft fatigue, or occlusion, as well as to assess and quantify the aortoiliac anatomo-morphological changes over time. In cases where adverse outcomes were clinically suspected or indicated by cDUS during follow-up, interim CTAs were performed. Follow-up beyond the 5th postoperative year continued using the same principles. All follow-up spiral iodine-enhanced CTAs were performed at the same laboratory, without oral gastrografin intake and with a slice thickness of 0.5 mm and an interval of 0.3 mm. Post-imaging processing was based on analysis of Picture Archiving and Communication System (PACS) data, which were collected prospectively in an electronic database and were analyzed retrospectively using the central lumen line technique [13] and the curved path reformation tool (3D curved MPR) from Horos^TM^ (Horosproject.org, sponsored by Nimble Co LLC d/b/a Purview in Annapolis, MD, USA) software v.4.0. As no external core laboratory was available to validate the CTA measurements, each measurement was independently performed twice by two blinded investigators (A.G.P. and D.A.C.) to minimize potential bias. After intra- and inter-observer variability analysis, the final value of each anatomical variable studied was calculated as the average of the four values resulting from the two blind measurements of the two observers. Our protocol stipulated that all potential differences >10% between the two observers in any of the variables should be settled by a third senior investigator (G.A.P.), and his blinded measurement would serve as the final mean value.

### 2.1. Definitions of Post-Imaging Data

Routine CTA analysis included the identification of any type of endoleak or migration. Considering stent graft migrations, we identified two types: first, the proximal landing zone migration (PLZM), which was defined as the caudal displacement of the endograft ≥5 mm compared with its initial position in 1st month’s CTA, and second, the distal landing zone migration (DLZM), defined as the cephalad retraction of the endograft limb(s) ≥10 mm from their original position in the 1st month’s CTA [14]. The concomitant presence of type Ia, Ib, III, or IV endoleaks with any of the aforementioned migration types was considered an absolute indication for immediate re-intervention. Additionally, an isolated PLZM was considered clinically important and was considered an absolute indication for re-intervention if the caudal displacement of the endograft exceeded ≥10 mm. The same threshold was also used for secondary intervention in cases of cephalad retraction of limb(s) in DLZM. Routine CTA follow-up included also the measurement of the maximum AAA sac diameter. AAAs were considered stable if changes in maximum diameter were ≤±5 mm, while the definition of sac regression was based on the detection of a decrease in maximum diameter >5 mm.

**Figure 1:**
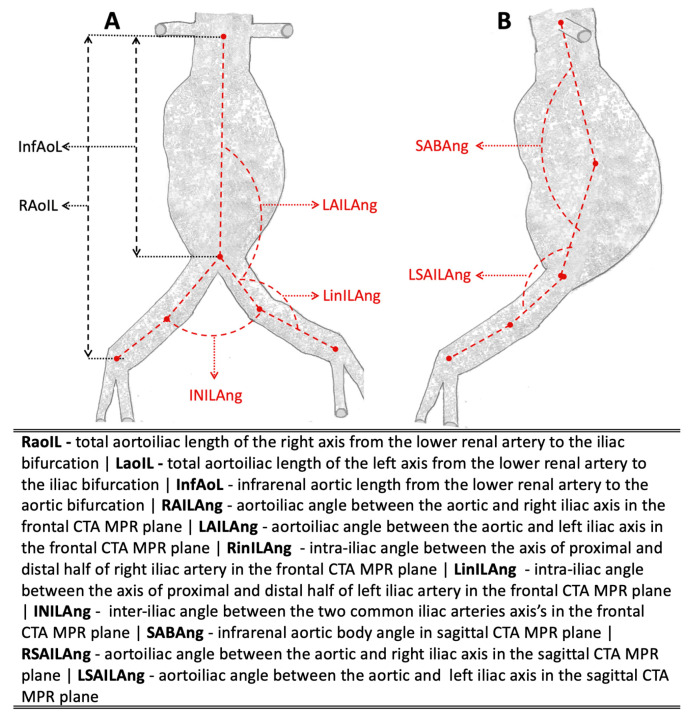
CTA measurement protocol of the 11 aortoiliac geometric primary variables: (**A**) aortic lengths and angles in the frontal plane CTA MPR and (**B**) angles in the left sagittal plane CTA MPR. The first letter R or L in the abbreviations indicates the right or left side, respectively, in bilateral variables.

In addition to routine CTA measurements, this study specifically analyzed a total of eleven aortoiliac geometrical primary variables, as illustrated in Figure 1. In addition, to investigate the role of potential factors that may have influenced the long-term aortoiliac geometrical alterations and their potential association with the clinical outcomes, we calculated, as secondary variables, the percentage difference of the 11 primary variables between the time intervals of the study. The secondary variables in cases of bilateral measurements were calculated as the mean value between the right- and left-side percentage difference.

The primary clinical endpoint was considered to be the occurrence of any complication related to the graft placement or reintervention (ARC). ARC included the following adverse events: clinically significant endoleaks (in terms of type Ia, Ib, III, or IV endoleaks or type II endoleaks with the AAA’s sac growing), migrations (PLZM, DLZM, or both types), stent graft limb occlusions, and all mortality events that were related to the index EVAR procedures or reinterventions for the treatment of ARCs. Additionally, we recognized 2 secondary endpoints: The 1st was all documented migration cases, and the 2nd was the failure of AAA sac regression. Considering patients who developed adverse events necessitating reintervention or reoperation during the follow-up period, appropriate treatment was provided in a timely manner. All clinical decisions and subsequent interventions were made and performed by the primary operating surgeon responsible for the index EVAR procedure.

### 2.2. Statistical Analysis

A detailed description of the statistical methods used in this study is available in the Supplementary Materials File S1.

## 3. Results

**Figure 2:**
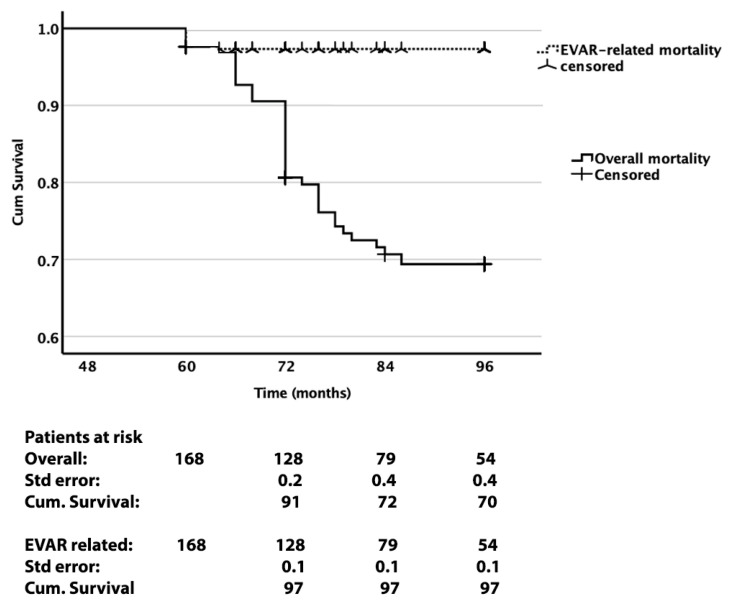
Kaplan–Meier survival analysis of overall and EVAR-related mortality at 8 years.

Sixty-three patients were excluded, as they were lost to follow-up, and finally, 168 patients were enrolled in the study protocol. Demographics, details on the risk factors, 30-day perioperative data, and details of patient exclusion are presented in Table 1. The 30-day primary technical success was 100%. The median–IQR age of the entire cohort was 72.0–9.0 years, and 6 patients were women. A total of 61% of the patients were under antiplatelet medication, consistent with standard secondary prevention practices. Additionally, 42% and 9.5% of the enrolled patients suffered from coronary and peripheral artery disease, respectively. The median–IQR duration of follow-up was 77–24.0 months (min 60 and max 96 months). The overall and EVAR-related mortality rates were 23.8% and 2.4%, respectively. Figure 2 shows the relative Kaplan–Meier curves and survival analysis.

**Figure 3:**
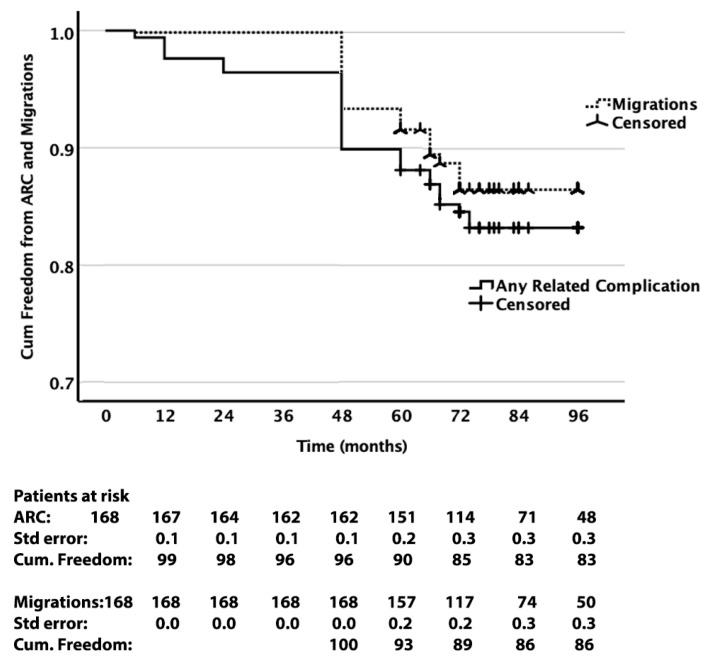
Freedom from any index EVAR or reintervention-related complications (ARC) and from migrations at 8 years.

A total of 27 (16.1%) ARC events occurred during follow-up. In 21 cases, an endograft migration was detected. In 20 of those cases, a combination of migration with clinically significant endoleak was detected. Table 2 summarizes the clinical outcomes at the 1-, 2-, and 5-year follow-up mark, and the total events that occurred for the entire follow-up period. Figure 2 shows the Kaplan–Meier survival analysis of ARC and migration endpoints. Figure 3 depicts the Kaplan–Meier curve of freedom from ARC and migration. It is noteworthy that approximately 30% of sacs growing endoleaks and migrations were detected during the period after the 60th month of follow-up. The mean ± stdv preoperative maximum AAA diameter was 57 ± 3.0 mm (min 48–max 64), and the respective postoperative values at 60 months were 50 ± 10 mm (min 30–max 72).

Table 3 shows the univariate analysis of total follow-up clinical outcomes in relation to the type and characteristics of the implanted stent grafts. The incidence of primary and secondary endpoints was significantly different between the six implanted aortic endografts. The suprarenal proximal fixation with hooks (n = 105) was significantly associated with better outcomes in terms of any related complications and migrations, and with better AAA sac regression rates, compared with those with infrarenal fixation or suprarenal fixation without hooks (n = 63). Additionally, endografts with Dacron liner material showed significantly higher sac regression rates (64.6%) compared with the ePTFE endografts (31.7%). However, the multinomial logistic regression analysis (Supplementary Table S1) confirmed that the suprarenal fixation with hooks was the only independent factor, which was still associated with better outcomes, showing a *p* value < 0.001 for all primary and secondary endpoints, and a seven-fold increase in the likelihood of aneurysm sac regression.

### 3.1. Aortoiliac Geometrical Remodeling

#### 3.1.1. Lengths

**Figure 4:**
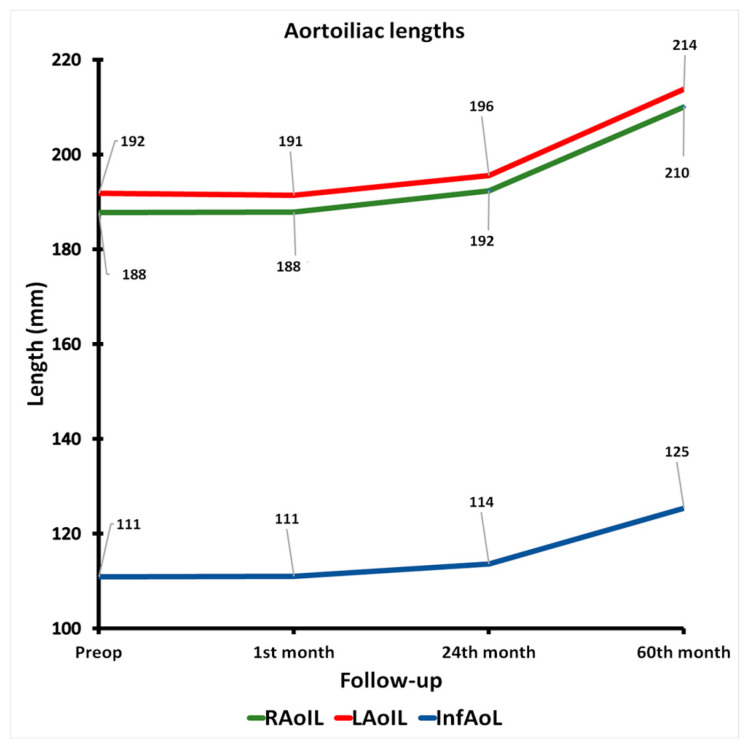
Graph of evolution in aortoiliac lengths, from preoperative to 60th-month CTA. (RAoIL = right total aortoiliac length, LAoIL = left total aortoiliac length, InfAoL = infrarenal aortic length).

The total aortoiliac lengths (RAoIL and LAoIL, Supplementary Table S2) increased slightly by a mean of 2.2% in the first two postoperative years and with an increased rate in the following 3 years, reaching an overall mean increase of 11.5%. The respective observed rates of progression in the length of the infrarenal aorta (InfAoL, Supplementary Table S2) were similar and estimated at 2.4% and 12.9%, respectively. Figure 4 showcases the length increase of the infrarenal aorta and aortoiliac axis.

#### 3.1.2. Angulations

**Figure 5:**
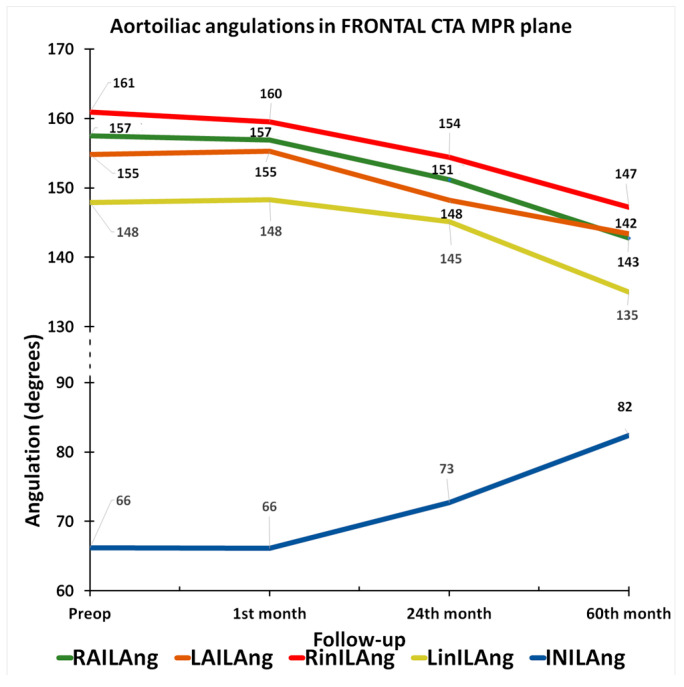
Graph of evolution in aortoiliac angulations in frontal CTA MPR plane, from the preoperative to 60th-month CTA. (RAILAng = right aortoiliac angle, LAILAng = left aortoiliac angle, RinILAng = right intra-iliac angle, LinILAng = left intra-iliac angle, INILAng = inter-iliac angle).

**Figure 6:**
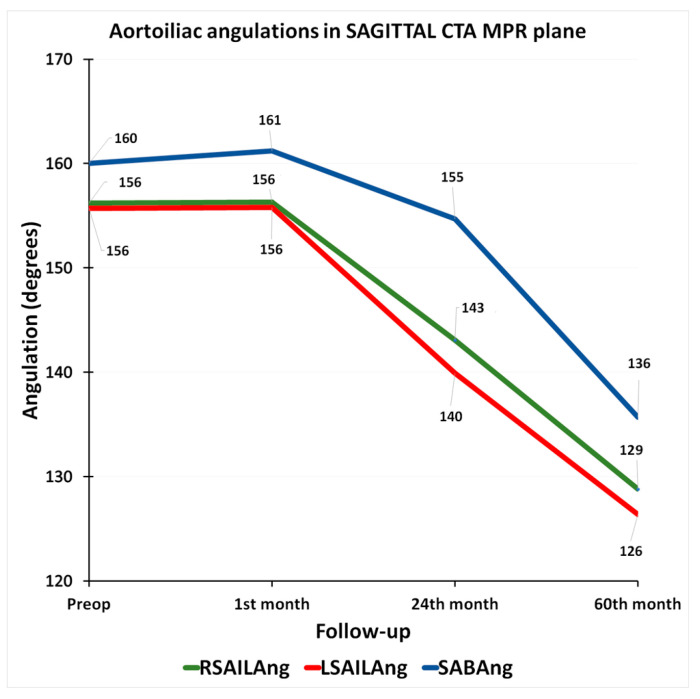
Graph of evolution of aortoiliac angulations in sagittal CTA MPR plane, from preoperative to 60th-month CTA. (RSAILAng = right sagittal aortoiliac angle, LSAILAng = left sagittal aortoiliac angle, SABAng = sagittal infrarenal aortic body angle).

Considering right and left aortoiliac angulations (RAILAng and LAILAng, Supplementary Table S2) measured in the frontal CTA MPR plane, we recorded a mean decrease of 4.0% in the first 2 years and an overall mean decrease of 8.3% at 5 years (Figure 5), while the respective mean rates of progression in the intra-iliac (RinILAng and LinILAng, Supplementary Table S2) angles were −2.9% and −8.3%. A notably greater progression rate was documented in the inter-iliac (INILAng, Supplementary Table S2) angle, which increased by 10.2% at 2 years and by 24.9% at 5 years (Figure 5). The measurement of angulations in the sagittal CTA MPR plane revealed a mean decrease in aortoiliac angulations (RSAILAng and LSAILAng, Supplementary Table S2, Figure 6) of −9.2% at 2 years and of −18.2% at 5 years. The mean progression rates in infrarenal aortic body angle (SABAng, Supplementary Table S2) were −3.3% and −15.1%, respectively.

#### 3.1.3. Relation of Demographics and Endografts to Remodeling

Univariate analysis of the potential association of age greater than 72 years (median age of series) and history of hypertension, diabetes, chronic obstructive pulmonary disease, and statin medication with the percentage difference between the CTA measurements of the 1st and 60th months failed to show any statistically significant correlation (Table 4). Additionally, no relationship was found between the excess mean percentage difference in CTA measurements and the brand of endograft implemented (Table 4).

#### 3.1.4. Impact of Aortoiliac Geometrical Remodeling in Outcomes

Univariate analysis (Table 5) of the potential association between the progression of the measured geometric variables and the clinical outcomes revealed that no angulations were related and conversely, all length measurements were significantly associated with migrations but not with AAA sac regression failures. The same analysis was performed between all geometric variables and the characteristics of the implanted endografts, which showed a similar association (Table 5) between suprarenal fixation with hooks and the progression of all measured length variables. In addition, the observed evolution of angulation variables was not correlated with suprarenal fixation of endografts with hooks. The same findings were observed when comparing the lining material with all geometric variables.

The mean aortoiliac length progression rates, which were calculated as the mean value between the right and left total aortoiliac length rates, and the infrarenal aortic length progression rates, were entered in a multivariate logistic regression model (Table 6) along with the suprarenal fixation with hooks to investigate their potential independent association with endograft migrations and failures of AAA sac regression. In this multivariate model, the remodeling of length variables failed to show an association with clinical outcomes, in contrast with the suprarenal fixation with hooks, which was confirmed to be the only independent factor associated with fewer endograft migrations and with a 5.4-fold increase in the possibility of AAA sac regression.

Table 7 presents the results of the linear regression analysis, in which the dependent variable was the change in maximum AAA diameter between the preoperative and 60th-month CTAs. The percentage changes in geometrical remodeling variables between the 1st and 60th months served as potential predictors. The analysis demonstrated that excessive remodeling of both the aortoiliac lengths and the infrarenal aortic length was significantly associated with reduced AAA sac regression rates (*p* < 0.001 for both variables). In contrast, remodeling of the angulation parameters did not show any significant predictive value for sac regression.

## 4. Discussion

The current literature on aortoiliac remodeling after EVAR has primarily focused on changes in the proximal aortic neck [5,7,8,14,15,16,17]. This is the first study with prospectively collected data focusing on post-EVAR long-term aortoiliac remodeling in terms of lengths and angulations, as well as on its clinical implications after the endovascular treatment of AAAs utilizing exclusively contemporary bifurcated aortic grafts.

Our findings demonstrate that extensive and progressive aortoiliac remodeling occurs over time, even after a technically successful EVAR. Specifically, we observed an average 11.5% increase in total aortoiliac vessel length and a 12.7% lengthening of the infrarenal abdominal aorta, which appeared to be related to lower sac regression rates. Notably, the vessel elongation occurred at a rate of approximately 1% annually during the first two years, accelerating to over 3% annually during years three to five, indicating a continued remodeling trajectory beyond five years. Angulation also changed significantly: The angle between the common iliac arteries increased by nearly 25%, while most other measured angles decreased. The most pronounced change was a 15% reduction in the sagittal angulation of the infrarenal aortic body. Collectively, these findings depict a remodeling characterized by elongation of the aortoiliac axis with simultaneous movement of the infrarenal aortic body to a more posterior position, with a significantly increased angulation of its body and a lateral divergence of the iliac arteries. Notably, this remodeling appears to be linear during the first 2 years and quadratic or cubic afterwards, expanding even after the 5-year follow up mark. This finding highlights the clinical importance of a strict long-term follow-up. Although no definitive clinical recommendations can be made regarding the anatomical features and remodeling of the infrarenal aorta in relation to the EVAR procedure, evidence from our study, as well as from other studies, demonstrates that long-term aortic remodeling does occur. This remodeling appears to influence long-term clinical outcomes, particularly in terms of endoleaks and graft migration, affecting both the proximal neck and distal landing zones [18,19].

The observed anatomical alterations are the results of three main factors. The first is the progression of aneurysmal disease, which has been suggested to continuously affect the aortoiliac vessel wall in the same manner as the one leading to the expansion of the aorta and the development of AAAs in the first place [17,20,21,22]. Additionally, recent histological evidence indicates that upon the implantation of the endoprosthesis a hypoxic response occurs in the arterial wall, resulting in hypertrophic scarring of the vessel wall and leading to further weakening of the deformed aorta [23]. The second factor is the altered yet continuously acting hemodynamic forces following EVAR and, in particular, the oscillatory wall shear stress in the arteries, which is usually concomitantly affected by atherosclerosis [23,24,25]. The third factor is related to the type and sizing of the implanted endograft and involves several parameters, such as the radial forces exerted by the metal skeleton on key “pressure” points at sealing zones or at the aortic bifurcation, the extent of graft oversizing, and, finally, the length of the uncovered parts of the aortoiliac vessels at the proximal and distal sealing zones [21,26].

Regarding the recognition of potential predisposing factors of aortoiliac remodeling, Chandra et al. [11], in the only available up-to-date study involving 88 patients treated with now-outdated endografts, reported a possible univariate association of aortoiliac elongation with the presence of chronic obstructive pulmonary disease and the use of statins. However, this suggested association was not confirmed in our analysis, which included a significantly larger cohort.

To evaluate the clinical relevance of remodeling, we used “all related complications” (ARC) as our primary endpoint and considered “all migrations” and “failure of sac regression” as secondary endpoints. Our univariate analysis revealed a potential association between aortoiliac remodeling and device migration. Additionally, suprarenal proximal aortic graft fixation with hooks was associated with improved outcomes across all primary and secondary endpoints, as well as with reduced aortoiliac length remodeling. Multivariate analysis confirmed that suprarenal proximal fixation with hooks was the only predictor of AAA sac regression, with a seven-fold increased probability of regression in the isolated analysis of the stent graft’s properties and with a five-fold increased probability of regression when excessive aortoiliac length remodeling was entered in the equation. It is noteworthy that approximately one-third of the total migrations and endoleaks were observed in the period after the 60-month follow-up. This finding emphasizes the importance of CTA follow-up even after a seemingly successful EVAR procedure at 60 months.

As with all single-center observational studies [27], our research protocol has certain limitations that preclude establishing definitive cause-and-effect relationships. Another limitation of our research protocol is the potential for selection bias. Despite the considerable number of included patients, our cohort may still be insufficient to draw firm and robust conclusions. Furthermore, we included data from patients who were treated with various endografts differing in design and properties, which may have influenced the reported outcomes. It is also important to note that our data represent a single vascular surgeon’s experience and may not reflect a global reality. However, the study includes consecutive patients treated and followed up with using a common, standardized, and strict protocol, ensuring the greatest possible homogeneity and consistency of data. Additionally, all CTA measurements were performed in a fully blinded and methodologically objective manner. Ultimately, our aim was not to assess the aortoiliac remodeling with a single endograft but rather to evaluate this phenomenon across a range of devices in real-world clinical conditions. For all these reasons, our main finding regarding the effectiveness of proximal aortic fixation with hooks should not be interpreted as a recommendation against the use of other endografts with infrarenal fixation or with different design and properties. This is particularly relevant for proximal suprarenal fixation ePTFE stent grafts, which have been proven to be equally effective even for the treatment of AAAs with complex anatomy [28,29]. Instead, our findings support the conclusion that the three-piece endograft design featuring a nitinol exoskeleton, a Dacron fabric liner, and suprarenal fixation with hooks represents a durable platform of endografts for conventional EVAR that will be effective in the long term, as has been demonstrated in previous studies [30,31,32,33,34,35].

The ultimate take-home message from our study is that the long-term results of EVAR are seriously affected by the inevitable and continuous remodeling of the aortoiliac vessels, which is the reality to be considered in patient selection and EVAR planning. In this direction, it would be extremely interesting to investigate this phenomenon in conditions that further increase the fixation forces and stability of the stent graft at the level of the proximal infrarenal neck, as occurs with endostapling or fenestrated and branched EVAR techniques.

## 5. Conclusions

Our findings strongly support the idea that despite an initially successful EVAR, the underlying degenerative aneurysmal process continues to affect the aortoiliac vessels, resulting in significant aortoiliac geometrical remodeling over time. The extensive remodeling of aortoiliac length appears to be associated with lower AAA sac regression rates, whereas the suprarenal proximal aortic fixation with hooks may be a protective mechanism, potentially reducing the risk of adverse long-term outcomes. The long-term safety and efficacy of EVAR appear to require rigorous, meticulous, and potentially life-long follow-up, particularly for younger patients and those with greater life expectancy.

## Figures and Tables

**Table 1:** Demographics, risk factors, and 30-day perioperative data.

	Total n = 168 *
**Demographics**	
Age (years)	72.0–9.0
Male gender	162–96.4
**Preoperative clinical status**	
Hypertension	152–90.5
Diabetes	63–37.5
Dyslipidemia	147–87.5
Coronary artery disease	70–41.7
Chronic respiratory disease	19–11.3
Smoking	114–67.9
Peripheral arterial disease	16–9.5
Body mass index > 30	25–14.9
Antiplatelet	103–61.3
Statin	106–63.1
**30-day perioperative data**	
EVAR operative time (min)	68.0–25.0
EVAR fluoroscopy time (min)	11.0–6.0
Contrast medium (ml)	178.0–60.0
Locoregional anesthesia	159–94.6
30-day mortality/morbidity	0.0–0.0
30-day endoleak type I, III, or IV	0.0–0.0
30-day endoleak type II	16–9.5
Primary technical success	168–100.0

* Forty patients were lost to follow-up or died before the 60th month from unrelated causes after a fully successful EVAR. Eleven patients were excluded due to chronic renal failure and twelve because the implanted endograft was used in <10 total cases.

**Table 2:** Clinical follow-up data.

	1st Year	2nd Year	5th Year	Total Follow-Up
*n = 168*	(n—%)			
Endoleak type Ia	1–0.6	0	9–5.4	13–7.7
Endoleak type Ib	0	0	3–1.8	7–4.2
Endoleak type III or IV	0	0	1–0.6	1–0.6
Endoleak type II^SG^	0	2 ^–1.2	0	2–1.2
Total endoleaks type Ia, Ib, III, IV, II^SG^	1–0.6	2–1.2	13–7.7	23–13.7
Endoleak type II **^∝^**	11–6.5	12 ^–7.1	13–7.7	18–10.7
Migration at proximal aortic neck	0	0	7–4.2	10–6.0
Migration/retraction of iliac limbs	0	0	1–0.6	5–3.0
Both types of migration	0	0	6–3.6	6–3.6
Total migrations	0	0	14 ^#^–8.3	21–12.5
Rupture	0	0	3–1.8	3–1.8
Limb occlusion	3 *–1.8	0	0	3–1.8
Related reintervention	3–1.8	2–1.2	14 ^#^–8.3	25–14.9
Related mortality	0	0	4–2.4	4–2.4
All related complication events	4 *–2.4	2–1.2	14–8.3	27–16.1

II^SG^ = Type II endoleak with AAA sac growth. ^ Two of the new-onset type II endoleaks at 2 years required reintervention. **^∝^** = Total type II endoleaks evident in CTA at each time frame. * Two patients with limb occlusion also had a type II endoleak, and in one patient the limb occlusion was treated conservatively. ^#^ All migrations (2 with rupture) underwent reintervention.

**Table 3:** The 6 brands of implanted abdominal aortic grafts and univariate analysis of their potential association with follow-up outcomes.

		ARC ^		Migration *				Failure of Regression *^#^*		
Endografts	n—%	27–16.1	*p*		21–12.5		*p*		73–43.5	*p*
Endurant^TM^	58–34.5	6–10.3	**0.08**		5–8.6		**0.002**		18–31.0	**<0.001**
E-tegra^®^	28–16.7	2–7.1			1–3.6				6–21.4	
Treo^®^	19–11.3	2–10.5			1–5.3				5–26.3	
Anaconda^TM^	22–13.1	9–40.9			8–36.4				16–72.7	
AFX^®^ 2	29–17.3	7–24.1			6–20.7				23–79.3	
Ovation^TM^	12–7.1	1–8.3			0–0				5–41.7	
**Total**	168–100	27–16.1			21–12.5				73–43.5	
**Endograft features (n–%)**						**ARC ^ (27–16.1)**				
**Liner material**						**n—%**				***p***
ePTFE (41–24.4)						8–19.5				0.49
Dacron (127–75.6)						19–15.0				
**Type of proximal fixation**										
Suprarenal with hooks (105–62.5)						10–9.5				**0.003**
Infrarenal or suprarenal without hooks (63–37.5)						17–27.0				
						Migration * (21–12.5)				
**Liner material**						**n—%**				***p***
ePTFE (41–24.4)						6–14.6				0.63
Dacron (127–75.6)						15–11.8				
**Type of proximal fixation (n–%)**										
Suprarenal with hooks (105–62.5)						7–6.7				**0.003**
Infrarenal or suprarenal without hooks (63–37.5)						14–22.2				
						**Failure of regression ^#^ (73–43.5)**				
**Liner material**						**n—%**				***p***
ePTFE (41–24.4)						28–68.3				**<0.001**
Dacron (127–75.6)						45–35.4				
**Type of proximal fixation (n–%)**										
Suprarenal with hooks (105–62.5)						29–27.6				**<0.001**
Infrarenal or suprarenal without hooks (63–37.5)						44–69.8				

**^** ARC = Any complications related to index EVAR or secondary interventions. * Migration = migrations in proximal, distal, or both landing zones. ^#^ Failure of regression of the maximum diameter of the aneurysm’s sac. Significant differences with *p* value < 0.05 appears in bold.

**Table 4:** Analyzed effect of demographic factors and brand of endograft in percentage difference between the CTA measurements of the 1st and 60th months.

	1st vs. 60th %diff *	Age > 71 y *p*	Hypert ^#^ *p*	Diabetes *p*	COPD ^§^ *p*	Statin *p*	Graft *p*
Lengths							
Mean ^ AoIL	11.5 ± 4.5	0.518	0.729	0.210	0.895	0.185	0.147
InfAoL	12.9 ± 5.0	0.520	0.732	0.215	0.857	0.203	0.824
**Angulations in the frontal CTA MPR plane**							
Mean ^ AILAng	−8.3 ± 21.3	0.275	0.234	0.622	0.244	0.225	0.726
Mean ^ inILAng	−8.3 ± 23.4	0.921	0.894	0.717	0.091	0.085	0.578
INILAng	24.9 ± 10.0	0.461	0.269	0.676	0.391	0.658	0.580
**Angulations in the sagittal CTA MPR plane**							
Mean ^ SAILAng	−18.2 ± 19.1	0.274	0.234	0.629	0.231	0.214	0.664
SABAng	−15.1 ± 13.4	0.611	0.385	0.377	0.642	0.739	0.389

Analysis of the demographics was conducted by Mann–Whitney U-test and of the brand of endograft with Pearson’s chi-square test, using as variables in the rows the distribution above or below the mean percentage difference. * %diff = percentage difference between the CTA measurements of the 1st and 60th months. Hypert ^#^ = hypertension, ^§^ COPD = chronic obstructive pulmonary disease ^ Mean = the mean value of %diff between the two (right and left side) measurements.

**Table 5:** Independent samples T-test analyzing the relation of the suprarenal fixation with hooks and all types of endograft migrations with the percentage of evolution rate between CTA measurements of the 1st and 60th months. ^.

	Suprarenal Fixation with Hooks			All Migrations		
1 to 60 Months %diff *	Yes, n = 105	No, n = 63	*p*	Yes, n = 21	No, n = 147	*p*
*Lengths*						
RAoIL	11.1 ± 4.3	12.6 ± 4.8	**0.01**	13.1 ± 3.9	11.4 ± 4.6	**0.04**
LAoIL	10.9 ± 4.2	12.3 ± 4.8	**0.02**	12.8 ± 3.9	11.2 ± 4.5	**0.04**
InfAoL	12.2 ± 4.7	13.9 ± 5.3	**0.01**	14.5 ± 4.3	12.6 ± 5.0	**0.04**
**Angulations in the frontal CTA MPR plane**						
RAILAng	−10.6 ± 21.6	−7.0 ± 21.0	0.14	−3.1 ± 21.9	−10.1 ± 21.1	0.17
LAILAng	−8.8 ± 21.7	−5.0 ± 20.8	0.14	−1.12 ± 22.0	−8.2 ± 21.2	0.17
RinILAng	−8.6 ± 22.6	−7.6 ± 24.5	0.39	−11.9 ± 24.6	−7.7 ± 23.1	0.46
LinILAng	−8.7 ± 22.9	−7.6 ± 24.8	0.38	−12.0 ± 24.6	−7.9 ± 23.4	0.47
INILAng	24.9 ± 9.8	24.9 ± 10.3	0.49	26.6 ± 12.0	24.6 ± 9.7	0.47
**Angulations in the sagittal CTA MPR plane**						
RSAILAng	−18.7 ± 19.6	−15.5 ± 18.8	0.14	−12.0 ± 19.8	−18.3 ± 19.1	0.18
LSAILAng	−20.0 ± 19.2	−16.9 ± 18.4	0.14	−13.4 ± 19.4	−19.6 ± 18.7	0.18
SABAng	−15.0 ± 13.2	−15.4 ± 14.0	0.43	−19.5 ± 13.5	−14.5 ± 13.6	0.12

^ The same analysis was executed using as grouping variables the liner material and the aneurysm sac regression at 60 months, and it revealed non-significant differences between subgroups (*p* > 0.05 in all tests). * 1 to 60 months %diff = the percentage of evolution in measurements between the CTA of the 1st and 60th month of follow-up; all variables followed normal distribution. Significant differences with *p* value < 0.05 appear in bold.

**Table 6:** Multinomial logistic regression analysis of endograft characteristics with univariate positive association with primary and secondary endpoints.

	Sig. (p)	Exp (B)	95% C.I. for Exp (B)
			Lower–Upper
	Migrations * (n = 21–12.5%)		
Mean aortoiliac length ^§^, 1 to 60 months %diff ^	0.23	211.9	0.140–320.49
Infrarenal aortic length, 1 to 60 months %diff ^	0.22	0.059	0.001–5.679
Suprarenal fixation with hooks	**0.02**	0.308	0.113–0.843
	**Failure of Regression ^#^ (n = 73–43.5%)**		
Mean aortoiliac length 1 to 60 months %diff *	0.11	0.063	0.002–1.874
Infrarenal aortic length 1 to 60 months %diff *	0.10	12.331	0.571–266.19
Suprarenal fixation with hooks	**<0.001**	5.482	2.492–11.089

^§^ The value was calculated as the mean between the right and left total aortoiliac length. ^ 1 to 60 months %diff = the percentage of evolution in measurements between the CTA of the 1st and 60th months of follow-up. * Migration included proximal, distal, or both landing zone failures. ^#^ Failure of regression of the maximum diameter of the aneurysm’s sac. Significant differences with *p* value < 0.05 appear in bold.

**Table 7:** Linear regression analysis with dependent variables of the difference in maximum AAA diameter at 5 years ^ and as independent variables of the percentage of evolution in CTA measurements between the 1st and 60th months.

Percentage of Difference in CTA Measurements	*p* Value
Lengths	
Mean aortoiliac length *	**<0.001**
Infrarenal aortic length	**<0.001**
**Angulations in the frontal CTA plane**	
Mean aortoiliac angulation *	0.52
Mean intra-iliac angulation *	0.88
Interiliac angulation	0.12
**Angulations in the sagittal CTA plane**	
Mean aortoiliac angulation *	0.54
Infrarenal aortic body angulation	0.61

^ The mean ± stdv difference between preoperative and 60th-month maximum AAA diameter was 6.0 ± 9.0 mm (min −13 mm and max 20 mm). * In variables with bilateral measurements, the mean variable was calculated as the mean between right- and left-side values. Significant differences with *p* value < 0.05 appear in bold.

## Data Availability

Data are available by request from the corresponding author.

## Supplementary Materials

The following supporting information can be downloaded at: https://www.mdpi.com/article/10.3390/jcm14165626/s1, Table S1: Multinomial (binary) logistic regression analysis of endografts characteristics with univariate positive association with primary and secondary endpoints.; Table S2: Mean ± stdv values of CTA measurements of lengths and angulations analyzed by Wilcoxon Signed Ranks Test. File S1.
