# Supplementary material for: Long-Term Remodeling of Aortoiliac Vessels After Standard EVAR, the Reality to Be Considered
Source: J Clin Med. 2025 Aug 8;14(16):5626. doi: 10.3390/jcm14165626 (PMC12386428; doi:10.3390/jcm14165626)
Supplement: Supplementary file 1 [file jcm-14-05626-s001.zip › jcm-3770624-supplementary/jcm-3770624-supplementary/jcm-3770624-Supplementary File S1.pdf]

File S1. Statistical analysis.

All data were entered in a digital database and the statistical analysis including the assessment of inter- and intra-observers' variability by Bland-Altman plot and regression analysis [15] was performed using the IBM SPSS Statistics software - version 28.0 for iOS (IBM Corp, Armonk, NY, USA). All statistical tests applied were two-sided and the level of statistical significance level was set at p value <.05. The agreement between the blind measurements of the same observer as well between the two observers was proved excellent and the option of the third senior observer was not used in any of the included patients. Categorical variables are presented as counts and percentages and were analyzed using the chi-squared test. Distribution of continuous variables was explored with normality plots and Shapiro-Wilk test, and all primary variables were found to follow skewed distribution. In contrast, the secondary continuous variables were followed normal distribution. Continuous variables of demographics are presented as median and interquartile range. Continuous variables from CTA analysis were analyzed by non-parametric Wilcoxon Signed Ranks Test, as appropriate and in tables and text are presented as mean±stdv. Based on our patients and endografts inclusion, we identified as the only possible combination for proper statistical analysis the distinction of our series into 2 clinical subgroups, those with suprarenal fixation with hooks or not. Comparison of continuous secondary variables from CTA data between subgroups was based in 2 independent samples t-Students test and are presented as mean±stdv. Independent categorical variables with a positive univariate association with the 5-year clinical outcomes were further analyzed with multinomial (binomial) logistic regression analysis. In addition, the Kaplan-Meier analysis was used to construct survival plots and to assess the clinical outcomes during the follow-up. Finally, to evaluate the effects of remodeling continuous data in sac regression we used linear regression analysis.
