# Supplementary material for: Long-Term Remodeling of Aortoiliac Vessels After Standard EVAR, the Reality to Be Considered
Source: J Clin Med. 2025 Aug 8;14(16):5626. doi: 10.3390/jcm14165626 (PMC12386428; doi:10.3390/jcm14165626)
Supplement: Supplementary file 1 [file jcm-14-05626-s001.zip › jcm-3770624-supplementary/jcm-3770624-supplementary/Supplementary tables.pdf]

Supplementary Table S1. Multinomial (binary) logistic regression analysis of endografts characteristics with univariate positive association with primary and secondary endpoints.

	Sig. (p)	Exp (B)	95% C.I. for Exp (B) Lower - Upper
	ARC^ (n=27 – 16.1%)		
Liner material			
ePTFE (41 – 24.4) vs Dacron (127 – 75.6)	.07	.350	.111 – 1.104
Type of proximal fixation			
Suprarenal with hooks (n=105) vs Infrarenal or suprarenal without hooks	**<.001**	.152	.052 – .444
	Migrations (n=21 – 12.5%)*		
Liner material			
ePTFE (41 – 24.4) vs Dacron (127 – 75.6)	.054	.300	.088 – 1.023
Type of proximal fixation			
Suprarenal with hooks (n=105) vs Infrarenal or suprarenal without hooks	**<.001**	.125	.039 – .398
	Failure of Regression# (n=73 – 43.5%)		
Liner material			
ePTFE (41 – 24.4) vs Dacron (127 – 75.6)	.71	1.238	.394 – 3.894
Type of proximal fixation			
Suprarenal with hooks (n=105) vs Infrarenal or suprarenal without hooks	**<.001**	6.989	2.492 – 19.598

^ ARC = Any related with index EVAR or secondary interventions complication. * Migration included proximal, distal or both landing zones failures. # Failure of regression of the maximum diameter of the aneurysm's sac. Significant difference with p value <.05 appears bold-typed.

Supplementary Table S2. Mean ± stdv values of CTA measurements of lengths and angulations analyzed by Wilcoxon Signed Ranks Test.

	**Preop^**	**1st Month**	**24th Month**	**60th Month**	**1st vs 24th** ***p - %diff****	**1st vs 60th** ***p - %diff****
			Lengths			
RAoIL	187.7±21.1	187.9±21.2	192.3±22.2	210.0±27.2	<.001 – 2.3	<.001 – 11.6
LAoIL	191.8±22.4	191.4±22.4	195.6±23.3	213.8±28.0	<.001 – 2.1	<.001 – 11.4
	Mean percent evolution rate#				***2.2***	***11.5***
InfAoL	110.9±16.1	111.0±16.1	113.6±16.8	125.4±20.3	<.001 – **2.4**	<.001 – **12.9**
			Angulations in the frontal CTA MPR plane			
RAILAng	157.5±13.9	156.9±13.8	151.2±18.3	142.8±35.4	<.001 – (-3.9)	<.001 – (-9.3)
LAILAng	154.8±14.8	155.3±14.9	148.2±15.4	143.4±36.6	<.001 – (-4.1)	<.001 – (-7.4)
	Mean percent evolution rate#				***-4.0***	***-8.3***
RinILAng	160.9±18.0	159.5±17.9	154.4±20.9	147.2±38.3	<.001 – (-3.9)	<.001 – (-8.2)
LinILAng	147.9±18.0	148.3±18.0	145.1±19.2	135.0±36.7	<.001 – (-1.9)	<.001 – (-8.3)
	Mean percent evolution rate#				***-2.9***	***-8.3***
INILAng	66.2±11.5	66.1±11.5	72.7±12.6	82.4±14.8	<.001 – 10.2	<.001 – 24.9
			Angulations in the sagittal CTA MPR plane			
RSAILAng	156.2±12.3	156.3±12.3	143.1±13.7	128.8±31.7	<.001 – (-8.3)	<.001 – (-17.5)
LSAILAng	155.7±12.7	155.8±12.7	139.9±12.4	126.4±31.5	<.001 – (-10.1)	.003 – (-18.9)
	Mean percent evolution rate#				***-9.2***	***-18.2***
SABAng	160.0±11.2	161.2±11.4	154.7±13.5	135.7±22.3	<.001 – (**-3.3**)	<.001 – (**-15.1**)

^ Preop = preoperative, ***p - %diff:** p=p value of Wilcoxon Signed Ranks test between the CTA measurements at 1st, 24th and 60th month of follow-up and %diff = the percent difference between the CTA measurements at the same intervals, negative percentage of difference appears in parenthesis. #Mean value of %diff between the two (right and left side) measurements. %diff in single value variables and mean %diff in bilateral variables appears bold-typed.
